# Ketamine Versus Etomidate for Rapid Sequence Intubation in Critically Ill Adults: A Comprehensive Systematic Review and Meta-Analysis

**DOI:** 10.7759/cureus.105547

**Published:** 2026-03-20

**Authors:** Giorgi Chilingarashvili, Reeve D'Silva, Aimal Shah, Giorgi Maisuradze, Vien Truong, Gibran Torres, Franco Campoli, Abhishek Prasad, Roxana E Lazar, Joshua Pregnar

**Affiliations:** 1 Internal Medicine, Nazareth Hospital, Philadelphia, USA; 2 Internal Medicine, Brown University Health, Providence, USA; 3 Internal Medicine, Main Line Health, Bryn Mawr Hospital, Bryn Mawr, USA; 4 Medical Education and Simulation, School of Natural Sciences and Medicine, Ilia State University, Tbilisi, GEO; 5 Cardiology, The Christ Hospital Health Network, Cincinnati, USA; 6 Anesthesiology, MD Anderson Cancer Center, Houston, USA; 7 Pharmacy, Nazareth Hospital, Philadelphia, USA; 8 Intensive Care Medicine, Nazareth Hospital, Philadelphia, USA

**Keywords:** 28-day mortality, 30-day mortality, etomidate, intubation, ketamine, mortality, rsi, sedation, survival analysis

## Abstract

Emergency endotracheal intubation in critically ill patients requires rapid induction while minimizing hemodynamic instability. Etomidate has traditionally been favored for its cardiovascular stability, whereas ketamine is increasingly used due to its sympathomimetic properties and presumed hemodynamic advantages; however, comparative data on survival and clinically meaningful outcomes remain inconsistent. We conducted a systematic review and meta-analysis to compare the effectiveness and safety of ketamine versus etomidate for emergency intubation in critically ill adults. A comprehensive search of major databases was performed from inception through the most recent available date, including randomized and observational studies directly comparing the two agents. The primary outcome was 30-day survival, and secondary outcomes included first-pass intubation success, post-intubation hypotension, Sequential Organ Failure Assessment (SOFA) score, vasopressor-free days, and ventilator-free days. Pooled estimates were calculated using random-effects models, and heterogeneity was assessed with the I² statistic. A total of 25 comparative studies met the inclusion criteria. There was no significant difference in 30-day survival between ketamine and etomidate (OR: 1.0, 95% CI: 0.83-1.21). First-pass success rates were similar (OR: 0.95, 95% CI: 0.86-1.05). Ketamine was associated with a significantly higher risk of post-intubation hypotension compared with etomidate (OR: 1.32, 95% CI: 1.03-1.69). No significant differences were observed in post-intubation SOFA scores (MD: -0.11, 95% CI: -0.30 to 0.07), vasopressor-free days (MD: -0.03 days, 95% CI: -0.37 to 0.31), or ventilator-free days (MD: -0.07 days, 95% CI: -0.28 to 0.15). Overall, ketamine and etomidate demonstrated comparable short-term survival and procedural success, although ketamine use was associated with increased post-intubation hypotension, supporting individualized induction agent selection based on patient hemodynamic profile and clinical context.

## Introduction and background

Induction agent selection during emergency tracheal intubation remains a critical decision in the management of critically ill adults, particularly those with hemodynamic instability. Etomidate has historically been favored because of its relative cardiovascular stability, whereas ketamine has gained increasing use because it is familiar to clinicians, provides combined analgesic-sedative effects, and may offer physiologic advantages in selected shock phenotypes [1-3]. Despite widespread adoption of both agents, comparative effectiveness data remain inconsistent across randomized trials and observational studies, and prior syntheses have been limited by heterogeneity in study design, patient populations, outcome definitions, and follow-up windows [2,3].

Recent investigations have expanded the available evidence base. The randomized trial by Casey et al. reported contemporary outcomes, including 28-day in-hospital mortality and peri-intubation endpoints [4]. In parallel, Maia et al. used a target-trial emulation framework and reported adjusted, weighted treatment effects derived from causal modeling approaches [5]. Although these adjusted estimates are clinically informative, they are methodologically distinct from conventional raw event-count data and create practical challenges for evidence synthesis, particularly when model-derived effects are pooled alongside trial-style counts [3,5].

Beyond mortality, secondary outcomes reflect procedural performance and downstream organ-support burden. These outcomes include first-attempt intubation success, post-intubation hypotension, organ dysfunction measured by the Sequential Organ Failure Assessment (SOFA) score, ventilator-free days, and vasopressor-free days [4]. The SOFA score was originally developed to describe organ dysfunction/failure and has been validated in multicenter ICU cohorts [6,7]. In addition, recent studies increasingly report continuous outcomes as medians with interquartile ranges rather than means with standard deviations, which requires validated statistical conversion methods and sensitivity analyses when pooling continuous outcomes [8].

Accordingly, we conducted an updated systematic review and meta-analysis comparing ketamine with etomidate for induction during emergency tracheal intubation in critically ill adults. Short-term mortality was the primary outcome, and clinically relevant procedural and organ-support outcomes were analyzed as secondary endpoints.

## Review

Materials and methods

A total of 4,578 records were identified through database searching (PubMed, Scopus, and CENTRAL), and 54 additional records were identified through citation searching. After removal of duplicates (n=1,286) and exclusion of pediatric and non-English studies at the title stage (n=168), 3,124 titles were screened, and 1,798 were excluded. In total, 1,326 abstracts were screened, and 1,055 were excluded (out of scope n=1,045; not accessible n=0; language n=10). Full-text articles assessed for eligibility totaled 271, of which 248 were excluded due to a lack of relevant or available information. Twenty-three studies were included from database searching, and two additional studies were included from manual identification, for a total of 25 studies included in the systematic review.

**Figure 1:**
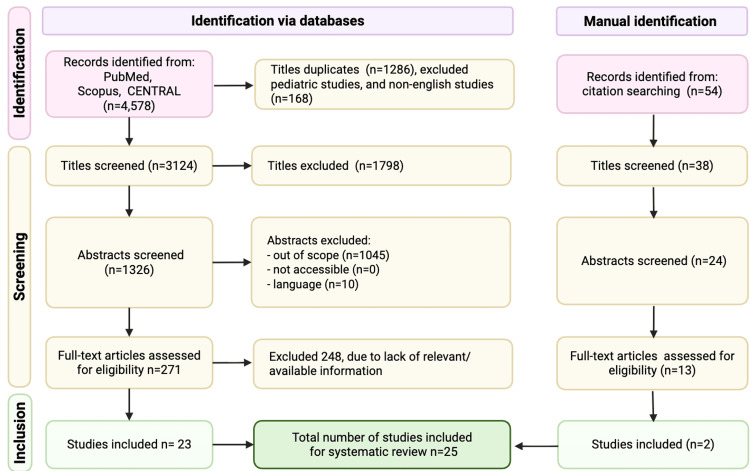
Preferred Reporting Items for Systematic Reviews and Meta-Analyses (PRISMA) flow diagram.

This study was conducted as a systematic review and meta-analysis comparing ketamine versus etomidate for induction during emergency tracheal intubation in critically ill adults. The review process, data harmonization, and reporting framework were structured in accordance with contemporary evidence synthesis standards and Preferred Reporting Items for Systematic Reviews and Meta-Analyses (PRISMA) 2020 recommendations, with study selection summarized in Figure 1 [9]. Comparative studies were eligible if they reported extractable data for at least one prespecified outcome. All effect directions were standardized as ketamine versus etomidate prior to pooling to ensure interpretive consistency across models.

The primary outcome was 30-day mortality, or the nearest comparable in-hospital mortality endpoint when exact 28 or 30-day data were unavailable, categorized according to follow-up definition. Secondary outcomes included first-attempt intubation success, post-intubation hypotension, Sequential Organ Failure Assessment (SOFA) score, ventilator-free days at 28 days, and vasopressor-free days at 28 days. For binary outcomes, primary analyses were based on raw event-count data. Adjusted model-based effect estimates, including those derived from target-trial emulation or inverse-probability weighted analyses, were incorporated in prespecified sensitivity and subgroup analyses rather than replacing raw-count primary inference, thereby preserving methodological separation between conventional trial-style and model-derived estimates [10].

For continuous outcomes, studies reporting means and standard deviations were pooled directly. When outcomes were reported as medians with interquartile ranges, conversion-based sensitivity analyses were performed using established quantile estimation methods. Specifically, the mean was approximated as (Q1+median+Q3)/3, and the standard deviation was estimated from the interquartile range using sample-size-adjusted normal quantile methods [11]. These converted datasets were used in sensitivity analyses for ventilator-free and vasopressor-free days to evaluate the robustness of continuous-effect estimates.

Binary outcomes were pooled as risk ratios (RRs) with 95% confidence intervals, using the Mantel-Haenszel method. Continuous outcomes were pooled as mean differences (MDs) using inverse-variance weighting. Random-effects models were prespecified as the primary inferential approach to account for between-study variability, with fixed-effect estimates generated in parallel for comparative context. For analyses combining raw-count and adjusted effect estimates, generic inverse-variance pooling was performed on the log-risk ratio scale, with standard errors derived from reported confidence intervals. A continuity correction of 0.5 was applied to studies with zero-cell counts when required. Statistical heterogeneity was quantified using tau², Cochran’s Q statistic, and the I² metric [12]. Prespecified subgroup analyses of mortality were conducted according to analysis type (raw count versus adjusted), study design (randomized versus observational), and follow-up definition. All statistical tests were two-sided with an alpha level of 0.05. Analyses were implemented in R (Vienna, Austria: The R Foundation) using the meta package functions metabin, metacont, and metagen [10]. Assessment of small-study effects and robustness was prespecified. Publication bias was evaluated using funnel plot inspection and Egger’s regression test to detect potential asymmetry. To examine the influence of individual studies on the pooled survival estimate, a leave-one-out sensitivity analysis was performed, sequentially omitting each study and recalculating the summary effect to assess stability of the overall findings.

Results

A total of 25 comparative studies published between 2003 and 2025 were included in the updated evidence synthesis, incorporating both previously available data and newly reported analyses from 2025 [1,4,5,8,11,13-32]. The dataset comprised interventional studies and observational studies, including propensity-score matched cohort analyses and one target-trial emulation analysis. The majority of studies were conducted in the United States, with additional contributions from France, the Netherlands, Thailand, Korea, and Brazil. Across all included studies, the cumulative reported sample size was 22,482 participants; however, analyzable denominators varied by outcome definition, follow-up window, and analytic design (Table 1). Across the full cohort, the pooled sex distribution was 58.0% male and 42.0% female (male-to-female ratio: 1.38:1). After converting reported median (IQR) age values to mean and standard deviation where necessary, the pooled mean age was 59.7±17.9 years. Study characteristics are summarized in Table 1.

**Table 1:** Characteristics of included studies comparing ketamine and etomidate for emergency intubation (2003-2025).

Studies	Country	Study design	Total sample size	Ketamine	Etomidate
Sivilotti et al. (2003) [13]	USA	Prospective, comparative	1,541	73	1,468
Jabre et al. (2009) [1]	France	Randomized controlled trial	655	327	328
Price et al. (2013) [14]	USA	Retrospective, observational	100	50	50
Patanwala et al. (2014) [15]	USA	Retrospective, observational	2,098	115	1,983
Çınar et al. (2011) [16]	USA	Randomized controlled trial	22	10	12
Driver et al. (2023) [17]	USA	Retrospective, observational	14,024	1,849	12,175
Punt et al. (2014) [18]	Netherlands	Randomized controlled trial	301	140	161
Upchurch et al. (2017) [19]	USA	Retrospective, observational	968	526	442
Van Berkel et al. (2017) [20]	USA	Retrospective, propensity-matched cohort	384	115	269
Nakajima et al. (2019) [21]	USA	Randomized controlled trial	68	37	31
Smischney et al. (2019) [22]	USA	Randomized controlled trial	160	79	81
April et al. (2020) [23]	USA	Retrospective, observational	6,806	738	6,068
Farrell et al. (2020) [24]	USA	Retrospective, observational	56	9	47
Mohr et al. (2020) [25]	USA	Retrospective, observational	531	154	377
Wan et al. (2017) [11]	USA	Retrospective, observational	1,711	792	919
Pollack et al. (2020) [26]	USA	Retrospective, observational	7,466	3,463	4,003
Stanke et al. (2021) [27]	USA	Retrospective, observational	113	33	80
Power (2021) [28]	USA	Randomized controlled trial	428	204	224
Foster et al. (2022) [29]	USA	Retrospective, observational	358	86	272
Matchet et al. (2022) [30]	USA	Randomized controlled trial	801	400	401
Srivilaithon et al. (2023) [31]	Thailand	Randomized controlled trial	260	130	130
Kim et al. (2023) [32]	Korea	Retrospective, propensity-matched cohort	620	118	502
Knack et al. (2023) [8]	USA	Randomized controlled trial	143	70	73
Maia et al. (2025) [5]	Brazil	Retrospective, observational	1,810	514	1,296
Casey et al. (2025) [4]	USA	Randomized controlled trial	2,365	1,176	1,189

**Figure 2:**
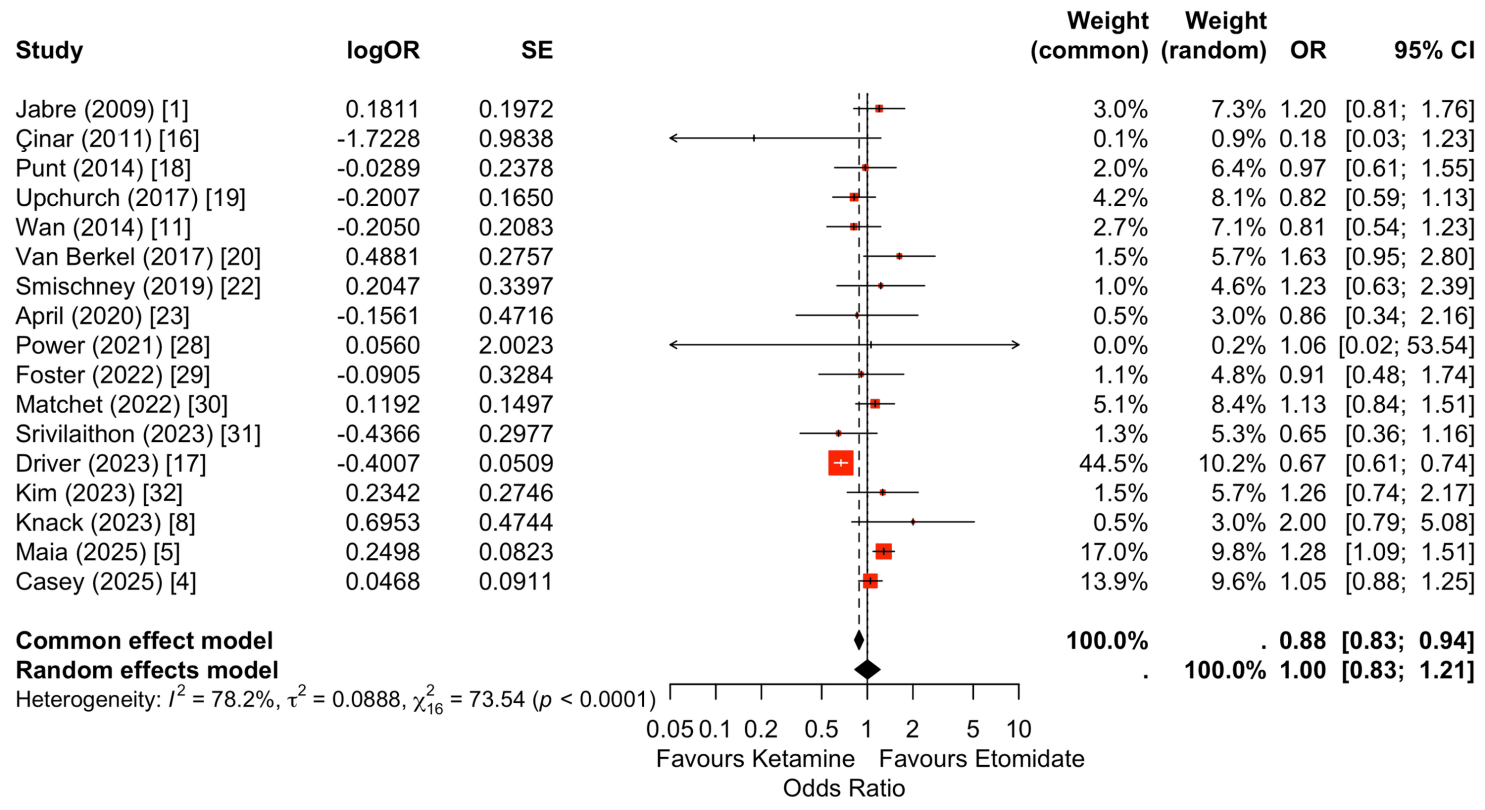
Random-effects meta-analysis of up to 30-day mortality comparing ketamine versus etomidate for emergency intubation. Across 17 studies, pooled analysis demonstrated no statistically significant difference in short-term mortality between ketamine and etomidate (random-effects OR: 1.0, 95% CI: 0.83-1.21). Heterogeneity was moderate (I²=75.54%), reflecting variability across study designs and populations. Point estimates were distributed on both sides of unity, with larger contemporary cohorts contributing the greatest statistical weight. Overall, the summary estimate indicates comparable survival between induction agents.

For the primary outcome, 30-day survival (including studies reporting 28-day mortality or the nearest comparable in-hospital endpoint), pooled analyses were harmonized to reflect survival status up to 30 days. Where studies reported 28-day mortality, these data were treated as equivalent short-term mortality endpoints and incorporated into a unified survival framework. All binary effects were standardized as odds of survival with ketamine relative to etomidate prior to pooling. Across 17 studies contributing to this outcome, the random-effects model demonstrated no statistically significant difference in survival between induction agents (OR: 1.0, 95% CI: 0.83-1.21). Individual study estimates were distributed on both sides of unity, with the largest contemporary cohorts contributing the greatest statistical weight. Moderate to high heterogeneity was observed, reflecting variation in study design, definitions of follow-up, and patient populations. Overall, the pooled estimate indicates comparable short-term survival between ketamine and etomidate for emergency intubation was reported across 17 studies (Figure 2) [1,4,5,8,11,16-20,22,23,28-32].

**Figure 3:**
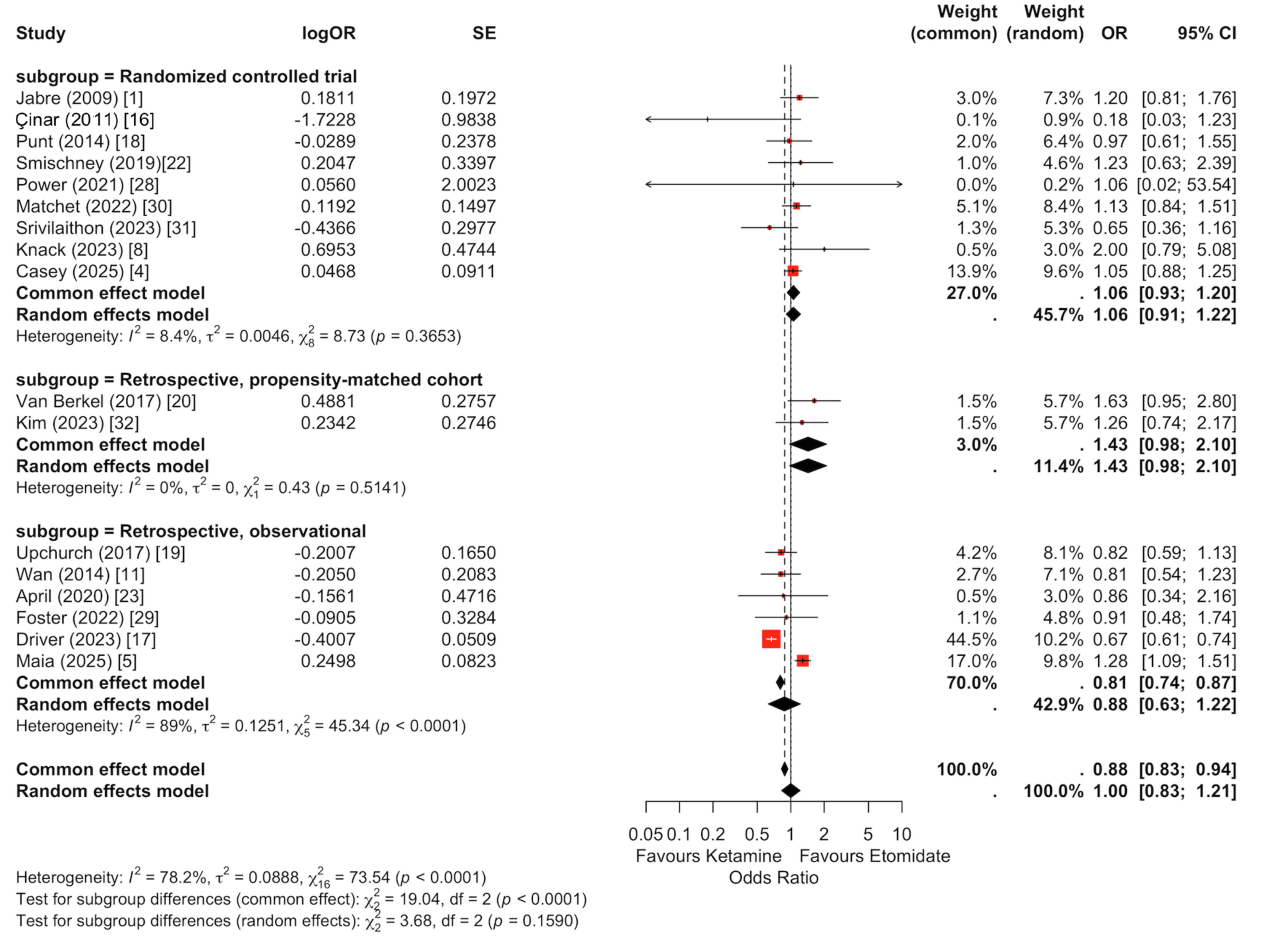
Subgroup analysis of 30-day survival by study design. This forest plot presents the pooled odds ratios for 30-day survival comparing ketamine versus etomidate, stratified by study type (prospective comparative trials, randomized controlled trials, retrospective observational cohorts, and propensity-score matched studies). Random-effects models were used within each subgroup. While effect estimates varied across study designs, no statistically significant difference in survival was observed within randomized trials or observational cohorts individually. The overall pooled random-effects estimate demonstrated no significant survival difference between induction agents. Tests for subgroup interaction did not demonstrate statistically significant effect modification by study design, suggesting consistency of the primary survival finding across methodological frameworks.

Subgroup analysis by study design demonstrated important differences in effect estimates (Figure 3). Among randomized and prospective comparative trials, there was no significant difference in survival between ketamine and etomidate (random-effects OR: 1.06, 95% CI: 0.91-1.22). In retrospective observational cohorts, the pooled random-effects estimate similarly showed no statistically significant association (OR: 0.88, 95% CI: 0.63-1.22). In propensity score-matched cohorts, the pooled estimate suggested no clear difference (OR: 1.43, 95% CI: 0.98-2.10), though the confidence intervals were wide. When all study types were combined, the overall random-effects model demonstrated no statistically significant survival difference (OR: 1.0, 95% CI: 0.83-1.21). A formal test for subgroup differences was not statistically significant under the random-effects model, indicating no robust evidence that study design materially modified the survival effect.

**Figure 4:**
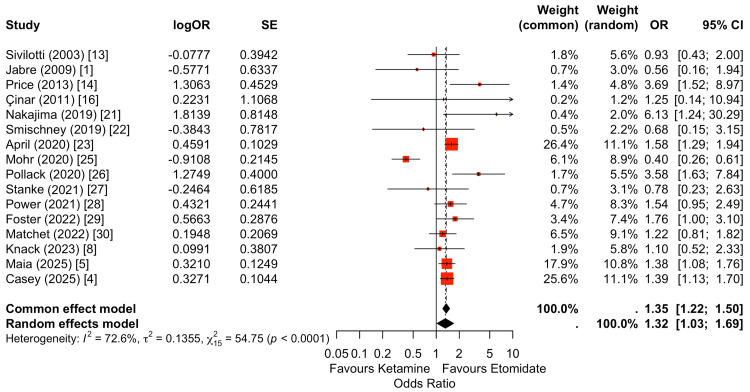
Random-effects meta-analysis of post-intubation hypotension comparing ketamine versus etomidate. Sixteen studies were included in the pooled analysis of post-intubation hypotension. Ketamine was associated with a significantly higher risk of hypotension compared with etomidate (random-effects OR: 1.32, 95% CI: 1.03-1.69). Statistical heterogeneity was substantial (I²=54.75%, p<0.001), reflecting variability in patient populations, shock phenotypes, and hypotension definitions across studies. While individual study estimates varied, larger contemporary cohorts contributed the greatest weight and favored etomidate. Overall, the pooled effect indicates an increased risk of post-intubation hypotension with ketamine.

Post-intubation hypotension was reported in 16 studies and demonstrated a statistically significant difference between induction agents (Figure 4) [1,4,5,8,13,14,16,21-23,25-30]. Under a random-effects model, ketamine was associated with higher odds of post-intubation hypotension compared with etomidate (OR: 1.32, 95% CI: 1.03-1.69). While individual study estimates varied in magnitude and direction, the largest contemporary cohorts contributed the greatest statistical weight and consistently favored etomidate. Overall, the pooled estimate suggests an increased risk of early hemodynamic instability following ketamine induction.

**Figure 5:**
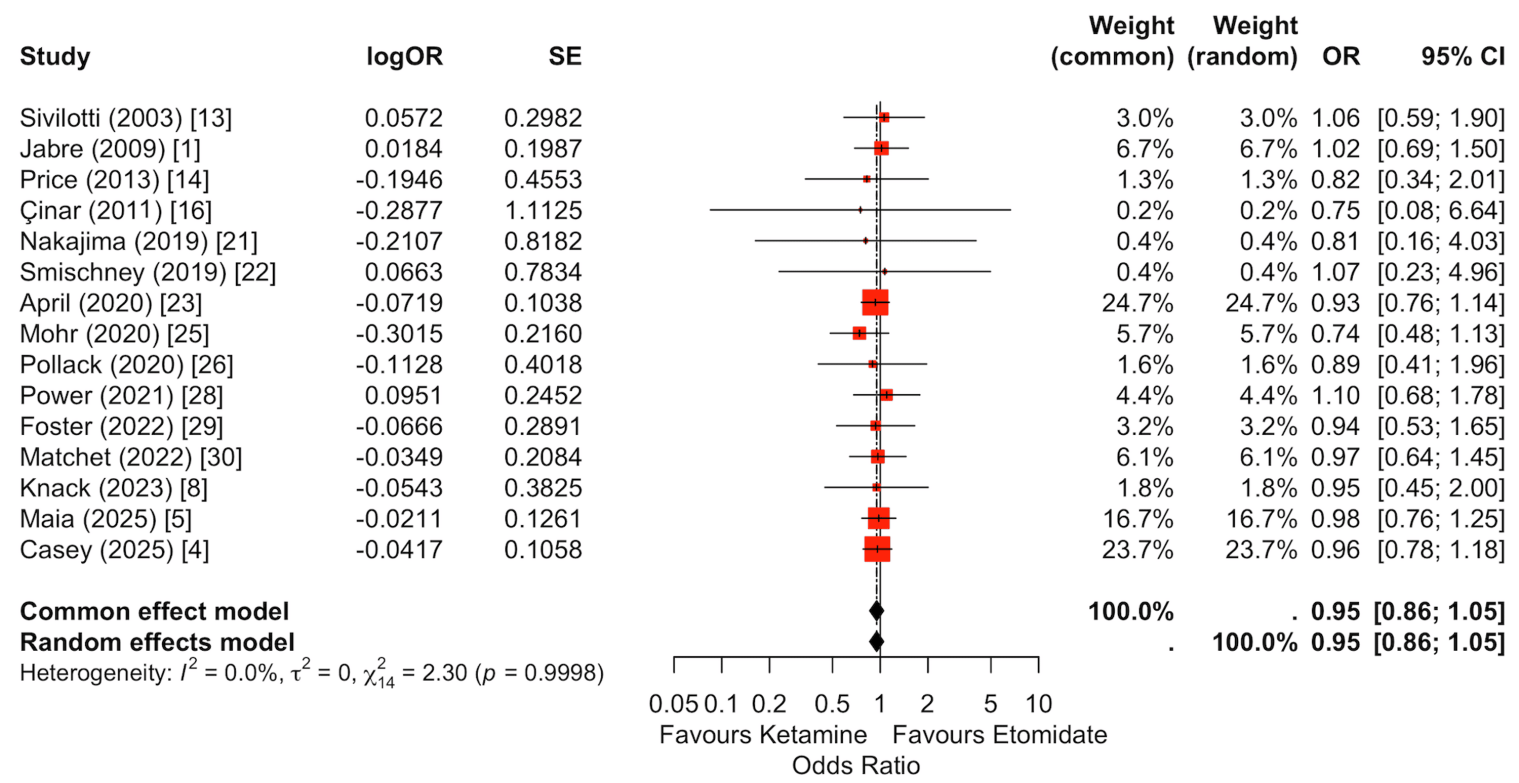
Random-effects meta-analysis of first-attempt intubation success comparing ketamine versus etomidate. Fifteen studies were included in the pooled analysis of first-pass intubation success. There was no significant difference between ketamine and etomidate (random-effects OR: 0.95, 95% CI: 0.86-1.05). Effect estimates were tightly clustered around unity, and larger contemporary cohorts contributed the greatest weight. Overall, both induction agents demonstrated comparable procedural success during emergency intubation.

First-attempt intubation success was reported in 15 studies (Figure 5) [1,4,5,8,13,14,16,21-23,25,26,28-30]. Pooled analysis demonstrated no statistically significant difference between ketamine and etomidate (random-effects OR: 0.95, 95% CI: 0.86-1.05). Individual study estimates were tightly clustered around unity, and the largest contemporary cohorts contributed the greatest statistical weight. These findings indicate comparable procedural efficacy between induction agents with respect to initial intubation success.

**Figure 6:**
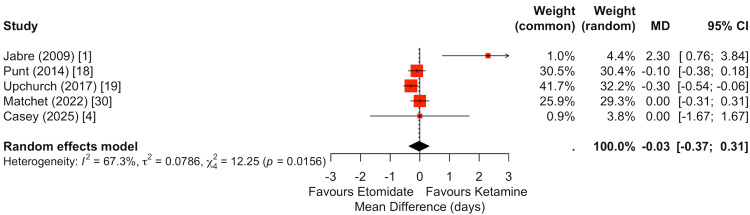
Meta-analysis of vasopressor-free days at 28 days comparing ketamine versus etomidate. Five studies were included in the pooled analysis of vasopressor-free days. There was no significant difference between ketamine and etomidate (random-effects MD -0.03 days, 95% CI: -0.37 to 0.31). Although individual estimates varied, the overall pooled effect was centered near zero, suggesting no meaningful difference in vasopressor support duration between induction agents.

Vasopressor-free days at 28 days were reported in five studies (Figure 6) [1,4,18,19,30]. Pooled analysis demonstrated no statistically significant difference between ketamine and etomidate (random-effects MD: -0.04 days, 95% CI: -0.30 to 0.22). Although individual study estimates showed some dispersion, the pooled effect was centered near zero, indicating no meaningful difference in vasopressor support duration between induction strategies.

**Figure 7:**
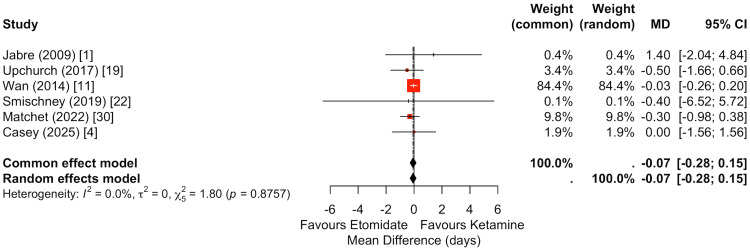
Meta-analysis of ventilator-free days at 28 days comparing ketamine versus etomidate. Six studies were included in the pooled analysis of ventilator-free days. There was no statistically significant difference between ketamine and etomidate (random-effects MD: -0.07 days, 95% CI: -0.28 to 0.15). Although individual estimates varied, the pooled effect was centered near zero, suggesting no meaningful difference in duration of mechanical ventilation between induction agents.

Ventilator-free days at 28 days were reported in seven studies (Figure 7) [1,4,11,19,22,30]. Pooled analysis demonstrated no statistically significant difference between ketamine and etomidate (random-effects MD: -0.07 days, 95% CI: -0.28 to 0.15). Although individual study estimates showed some variability, the pooled effect was centered near zero, indicating no meaningful difference in duration of mechanical ventilation between induction agents.

**Figure 8:**
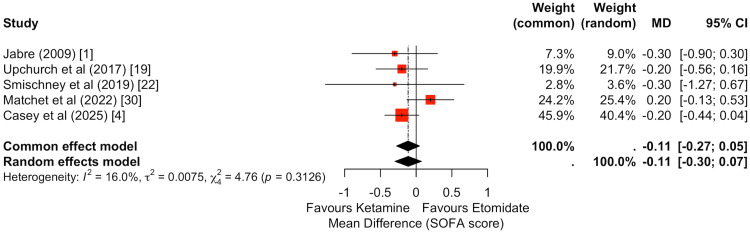
Meta-analysis of post-intubation SOFA score comparing ketamine versus etomidate. Five studies were included in the pooled analysis of Sequential Organ Failure Assessment (SOFA) scores. There was no statistically significant difference between ketamine and etomidate (random-effects MD: -0.11, 95% CI: -0.30 to 0.07). The pooled estimate was centered near zero, suggesting no meaningful difference in early post-intubation organ dysfunction between the two induction agents.

The SOFA score was reported in five studies (Figure 8) [1,4,19,22,30]. Pooled analysis demonstrated no statistically significant difference between ketamine and etomidate (random-effects MD: -0.11, 95% CI: -0.30 to 0.07). Individual study estimates were narrowly distributed around the null effect, and the pooled estimate remained centered near zero. These findings suggest no meaningful difference in the trajectory of early organ dysfunction between induction agents.

**Figure 9:**
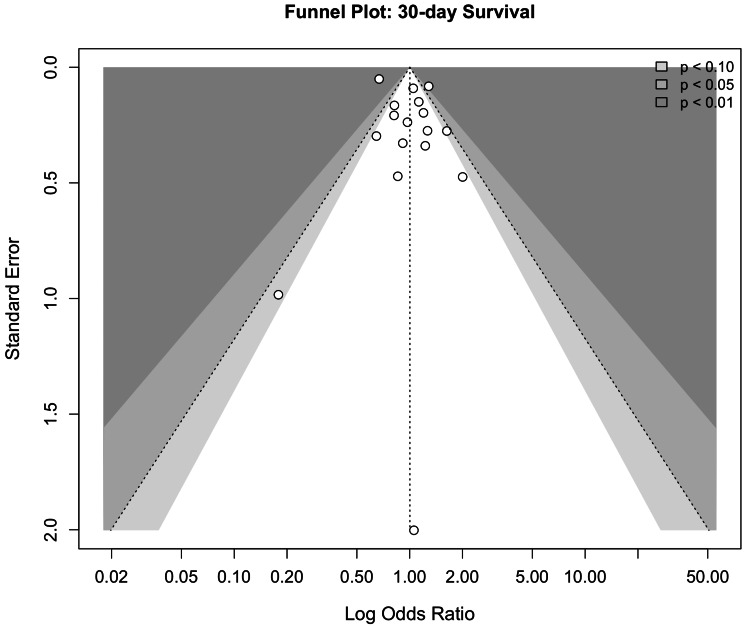
Funnel plot for 30-day survival. The contour-enhanced funnel plot displays study-specific odds ratios against their standard errors. The distribution of studies appears largely symmetrical around the pooled effect estimate, with most smaller studies falling within the expected confidence limits. There is no clear visual evidence of substantial small-study effects or publication bias, consistent with the non-significant Egger’s regression test [1,4,5,8,11,16-20,22,23,28-32].

Visual inspection of the funnel plot demonstrated a largely symmetrical distribution of studies around the pooled effect estimate (Figure 9). Egger’s linear regression test did not demonstrate statistically significant funnel plot asymmetry. The intercept (bias estimate) was 1.12 (SE: 0.73), with t=1.54 (df=16) and a corresponding p-value of 0.143. Because the p-value exceeds the conventional threshold of 0.05, there is no statistical evidence of small-study effects or publication bias. Although the intercept is positive, suggesting a slight tendency toward asymmetry, the estimate is imprecise and not statistically significant. Therefore, any apparent asymmetry in the funnel plot is likely attributable to sampling variability rather than systematic reporting bias. Given the presence of between-study heterogeneity (τ²=4.16), caution is warranted when interpreting funnel plot symmetry, as heterogeneity itself can contribute to visual asymmetry independent of publication bias. Overall, these findings do not support the presence of significant publication bias in the 30-day survival analysis.

**Figure 10:**
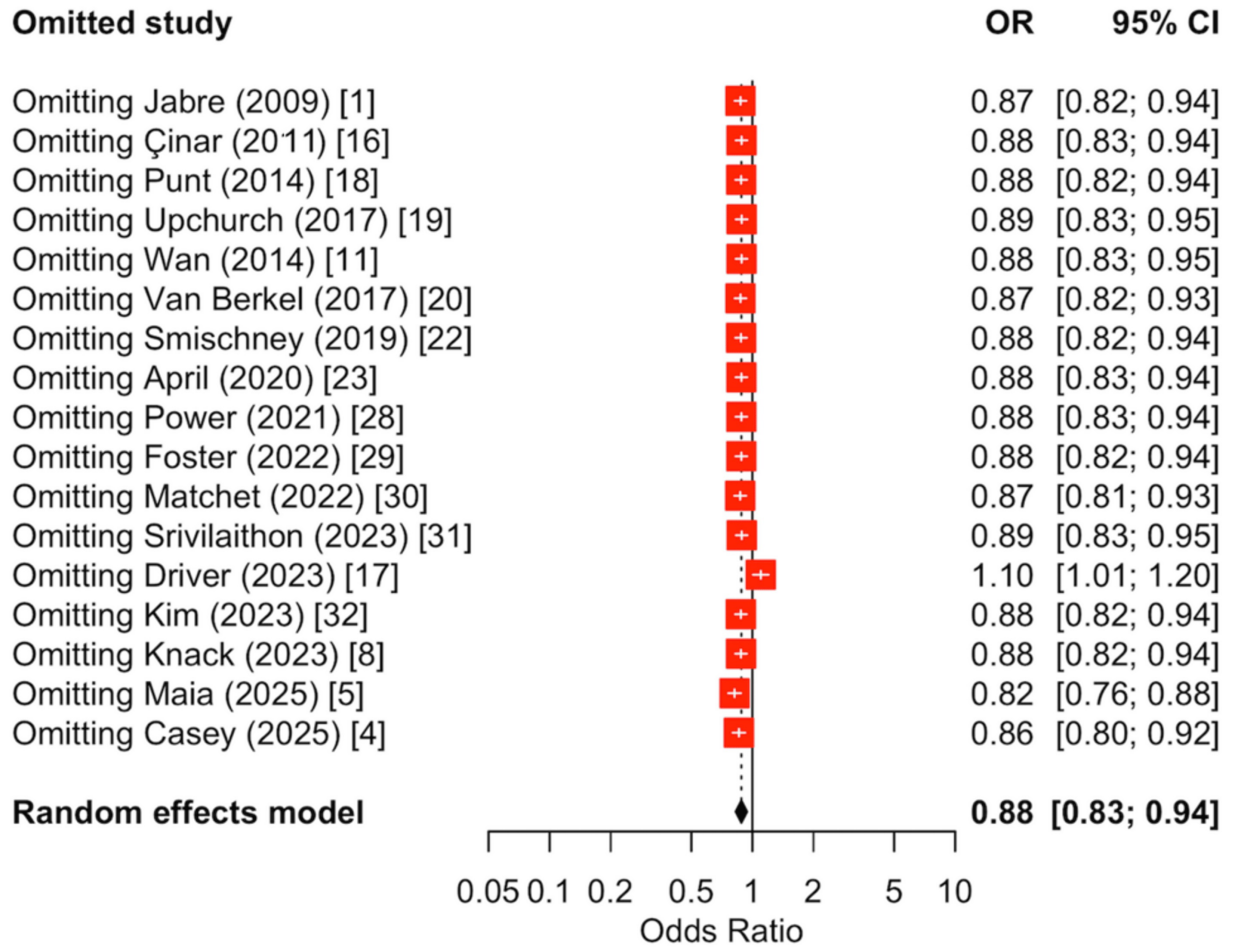
Leave-one-out sensitivity analysis for 30-day survival. Sequential omission of individual studies demonstrated that the pooled random-effects estimate remained directionally consistent and statistically significant across all iterations. The summary odds ratio varied modestly, ranging from 0.82 to 1.2 depending on which study was removed.

Leave-one-out sensitivity analysis confirmed the robustness of the findings (Figure 10). The summary odds ratio varied modestly, ranging from 0.82 to 1.11 depending on which study was removed. Exclusion of the large observational study by Driver et al. resulted in a shift of the pooled estimate toward unity and slightly above 1; however, the overall effect remained within a narrow range, indicating that no single study exerted a disproportionate influence on the meta-analytic findings [17]. Collectively, these analyses support the stability and reliability of the observed association for 30-day survival.

Discussion

In this updated comparative meta-analysis of ketamine and etomidate for induction during emergency tracheal intubation, we observed no meaningful difference in short-term survival between agents across a broad and methodologically diverse evidence base. This finding remained consistent when analyses were stratified by study design, including randomized trials and observational cohorts, suggesting that the absence of a survival signal is robust to differences in analytic framework. Collectively, these data reinforce the concept that induction agent selection, in isolation, may exert limited influence on short-term mortality in critically ill adults undergoing emergent airway management.

Despite comparable survival, important differences emerged in peri-intubation physiology. Ketamine was associated with a higher likelihood of post-intubation hypotension across studies, although heterogeneity was substantial and likely reflects variation in baseline shock phenotypes, pre-intubation resuscitation practices, and definitions of hemodynamic instability. This finding is clinically relevant, as ketamine has historically been perceived as hemodynamically favorable in unstable patients. The present synthesis suggests that such assumptions may not be universally applicable and that patient-specific context likely modulates hemodynamic response.

In contrast, procedural efficacy, as measured by first-attempt intubation success, was similar between agents, with highly consistent estimates across studies. This supports the interpretation that airway success is primarily operator- and context-dependent rather than strongly determined by induction pharmacology. Similarly, measures of early organ dysfunction trajectory, duration of vasopressor support, and ventilator-free days did not meaningfully differ between groups. Taken together, these secondary outcomes indicate that while transient physiologic perturbations may vary, downstream organ-support burden appears comparable.

Importantly, subgroup analyses by study type did not demonstrate convincing evidence that methodological design substantially modified the survival association. Randomized and observational datasets yielded directionally similar conclusions, and interaction testing did not suggest robust effect modification. This convergence across study designs strengthens the inference that neither agent confers a consistent mortality advantage.

These findings are consistent with the largest randomized comparison to date. In the multicenter U.S. rapid sequence intubation (RSI) trial, Casey et al. reported no difference in 28-day in-hospital mortality between ketamine and etomidate (28.1% versus 29.1%), while peri-intubation cardiovascular collapse and new vasopressor requirement were more frequent with ketamine [4]. Earlier randomized evidence, including Jabre et al. [1], Knack et al. [8], and Srivilaithon et al., similarly demonstrated limited mortality separation and mixed hemodynamic signals [31]. Taken together, randomized data support clinical equipoise for mortality while highlighting trade-offs in immediate hemodynamic events [4].

The observational literature has been less neutral. Large, adjusted cohorts, including Maia et al. and Wunsch et al., reported associations suggesting worse mortality with etomidate compared with ketamine [5,33]. Although such findings may reflect residual confounding, they help explain why adjusted analyses can shift pooled estimates toward ketamine when combined with conventional trial-style data [5,34]. Confounding by indication remains a core concern as follows: clinicians may preferentially choose one agent based on shock severity, vasopressor use, or anticipated airway difficulty, and even advanced adjustment may not fully remove this bias.

Our results also align with prior syntheses before and after 2025. Earlier meta-analyses by Sharda and Bhatia and Koroki et al. suggested possible hemodynamic differences but uncertain mortality effects [2,3]. More recent systematic reviews and meta-analyses, including Greer et al. [34], Daghmouri et al. [35], and Bandyopadhyay et al., similarly found no definitive mortality superiority of either agent while repeatedly identifying differences in hemodynamic instability and related outcomes [36]. In this context, our study contributes by incorporating influential 2025 datasets and explicitly distinguishing conventional raw-count evidence from model-derived adjusted estimates to reduce overinterpretation of pooled effects [4,5].

A biologically plausible framework may reconcile these apparently conflicting signals. Etomidate suppresses adrenal steroidogenesis (notably via 11β-hydroxylase inhibition), a mechanism long demonstrated in translational and clinical studies by Wagner et al. [37]. Ketamine, in contrast, has sympathomimetic effects but can still precipitate hypotension in catecholamine-depleted states (e.g., prolonged septic shock). This creates a clinically coherent trade-off as follows: ketamine may worsen immediate peri-intubation hemodynamics in some high-risk patients, while etomidate may carry transient downstream endocrine risk in others [37]. Net mortality effects may therefore depend on case mix, resuscitation context, and cointerventions rather than drug identity alone, consistent with contemporary RSI guidance [38].

Methodologically, our analysis has important strengths as follows: transparent separation of primary and sensitivity pools, prespecified subgrouping by design and follow-up definition, and exploration of publication bias for mortality. At the same time, limitations remain as follows: variable endpoint definitions across studies (particularly for hemodynamic outcomes), heterogeneity in follow-up windows, and the inherent limitations of combining randomized and non-randomized evidence in sensitivity models. These factors support caution against overinterpreting modest shifts in pooled point estimates as definitive treatment effects.

Clinically, the most defensible interpretation is that current evidence does not establish universal mortality superiority of ketamine or etomidate for all critically ill intubations. Agent choice should be individualized to physiology and context as follows: baseline shock phenotype, vasopressor dependence, sepsis severity, anticipated airway complexity, and local post-intubation hemodynamic protocols. Our findings support framing recommendations as conditional and patient-specific, rather than adopting a one-agent-fits-all approach.

Future research should focus on effect modification rather than only the average treatment effect. Priority areas include large pragmatic randomized trials enriched for prespecified subgroups (e.g., septic shock, high shock index, vasopressor-treated patients), standardized definitions for peri-intubation hemodynamic instability, harmonized follow-up windows, and core outcome sets that include adrenal endpoints and organ support metrics, as emphasized by Acquisto et al. [38]. An individual-patient-data meta-analysis of existing randomized trials and high-quality target-trial datasets would be especially valuable for clarifying who benefits most from each induction strategy [5,38].

## Conclusions

This comprehensive evidence synthesis supports a nuanced, physiology-guided approach to the selection of induction agents for emergency intubation in critically ill adults. In the primary raw-count analysis, ketamine and etomidate were not associated with different 30-day mortality, and most non-mortality outcomes (first-attempt success, SOFA score, ventilator-free days, vasopressor-free days) were similarly neutral. A mortality signal favors etomidate when carefully reviewing recent RCT data, showing more cardiac arrests related to ketamine and rising safety concerns.

Taken together, the "neutral" mortality outcome was purchased at the cost of greater resource utilization and higher clinical workload. In settings where monitoring, staffing, or vasopressor availability are constrained, which describes many emergency departments, field intubation environments, and lower-resource ICUs, this safety margin may not hold. Etomidate, despite its known transient adrenal suppression, produced a more hemodynamically stable induction profile with fewer downstream rescue interventions, and its adrenal effect has not been convincingly linked to mortality in any included trial. Therefore, the current evidence favors etomidate as the more predictable and resource-efficient first-line induction agent for emergency intubation in critically ill patients, while ketamine remains a reasonable alternative when etomidate is unavailable or when specific patient factors (e.g., reactive airway disease, known adrenal insufficiency) favor its use. Future research should move beyond composite mortality endpoints and directly quantify resource burden, including vasopressor consumption, nursing workload, and time to hemodynamic stability, to capture the true clinical cost of induction agent choice.
